# Modulation of Oligodendrocyte Differentiation by Mechanotransduction

**DOI:** 10.3389/fncel.2016.00277

**Published:** 2016-11-29

**Authors:** Tânia Lourenço, Mário Grãos

**Affiliations:** ^1^Biocant, Technology Transfer AssociationCantanhede, Portugal; ^2^Centre for Neuroscience and Cell Biology (CNC), University of CoimbraCoimbra, Portugal

**Keywords:** mechanotransduction, mechanobiology, oligodendrocyte, extracellular matrix, integrins, myelination, neural stem cells, differentiation

## Abstract

Oligodendrocytes (OLs) are responsible for the myelination of axons in the central nervous system (CNS). The differentiation of OLs encompasses several stages, through which cells undergo dramatic biochemical and morphological changes. OL differentiation is modulated by soluble factors (SFs)—such as growth factors and hormones—, known to be essential for each maturation stage. Besides SFs, insoluble factors such as extracellular matrix (ECM) proteins and other microenvironmental elements also play a pivotal role during OL differentiation. Recently, a growing number of studies were published concerning the effect of biophysical properties of the extracellular milieu on OL differentiation and myelination, showing the importance of ECM stiffness and topography, strain forces and spatial constraints. For instance, it was shown *in vitro* that OL differentiation and maturation is enhanced by substrates within the reported range of stiffness of the brain and that this effect is potentiated by the presence of merosin, whereas the myelination process is influenced by the diameter of axonal-like fibers. In this mini review article, we will discuss the effect of mechanical cues during OL differentiation and the possible molecular mechanisms involved in such regulation.

## Introduction

Oligodendrocytes (OLs) are specialized myelin-producing neural cells whose processes wrap around axons in the central nervous system (CNS). Myelin wrapping provides trophic support and insulation of axons, supporting structural and functional integrity of the neuronal networks present in the CNS, allowing for efficient saltatory conduction of action potentials (Michalski and Kothary, 2015). Primary demyelination is a pathologic condition with multiple possible causes, resulting in severe impairment of nerve impulse conduction in the CNS. When remyelination fails, axons and eventually neurons themselves degenerate progressively, causing impairment of CNS functions (Felts et al., 1997).

**Figure 1:**
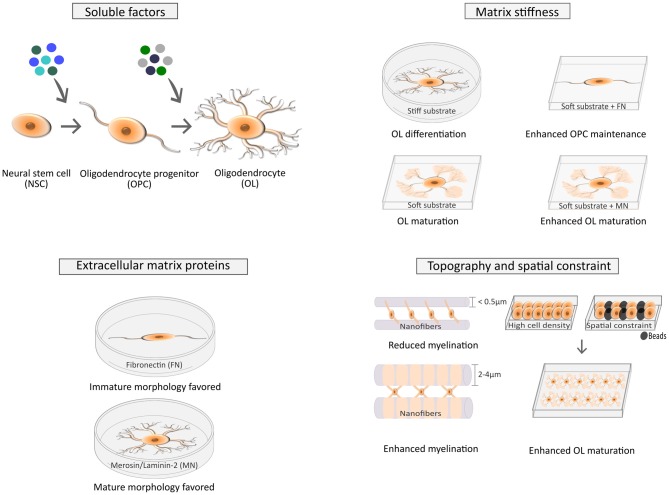
**Factors affecting cell fate of neural stem/progenitor cells (NSCs) and the differentiation of oligodendrocyte precursor cells (OPCs) into mature oligodendrocytes (OLs).** The fate of NSCs is influenced by several soluble factors (SFs; top left), as widely described in the literature (Rivera et al., 2010), but is also modulated by several biophysical cues (top right) such as stiffness (Keung et al., 2011, 2012) and the combination of strain and insoluble factors like extracellular matrix (ECM) proteins (Arulmoli et al., 2015). Maturation of OLs is also influenced by SFs (Baumann and Pham-Dinh, 2001; top left), ECM composition (Buttery and ffrench-Constant, 1999; Colognato et al., 2004; bottom left) and biophysical elements, like stiffness (Kippert et al., 2009; Jagielska et al., 2012; Lourenço et al., 2016; top right), spatial constraints and cell density (Rosenberg et al., 2008; Hernandez et al., 2016) and topography (Lee et al., 2012; Bechler et al., 2015; bottom right).

Several soluble factors (SFs), transcription factors and other biochemical elements were shown to play a pivotal role during the distinct developmental stages of the CNS, namely in what concerns the proliferation of oligodendrocyte precursor cells (OPCs), their migration and differentiation, and ultimately myelination of axons by mature OLs (Baumann and Pham-Dinh, 2001; Richardson et al., 2006; Bauer et al., 2009; Rivera et al., 2010; Michalski and Kothary, 2015). More recently, it was hypothesized that biophysical properties of the extracellular environment also play important roles during OL development (Bauer and ffrench-Constant, 2009; Kippert et al., 2009). Since then, several advancements were made (Figure 1) and together with mechanotransduction, mechanobiology of OLs has emerged as a vibrant field with important implications for fundamental and translational studies in the areas of OL biology and demyelinating disorders.

This mini-review article provides a concise overview of mechanotransduction, followed by a discussion of the mechanobiology of neural cells and OLs in particular.

## Principles of Mechanotransduction

Cells developed sensors for a variety of physical cues originating on the extracellular niche, such as shear stress, strain and other mechanical forces. Extracellular mechanical stimuli, including substrate stiffness (Engler et al., 2006; Fu et al., 2010), geometric constraints imposing cell shape (McBeath et al., 2004), and micro- or nano-topographic elements of the extracellular environment (Yim et al., 2010; Unadkat et al., 2011) can be converted into biochemical signals, hence the term mechanotransduction.

**Figure 2:**
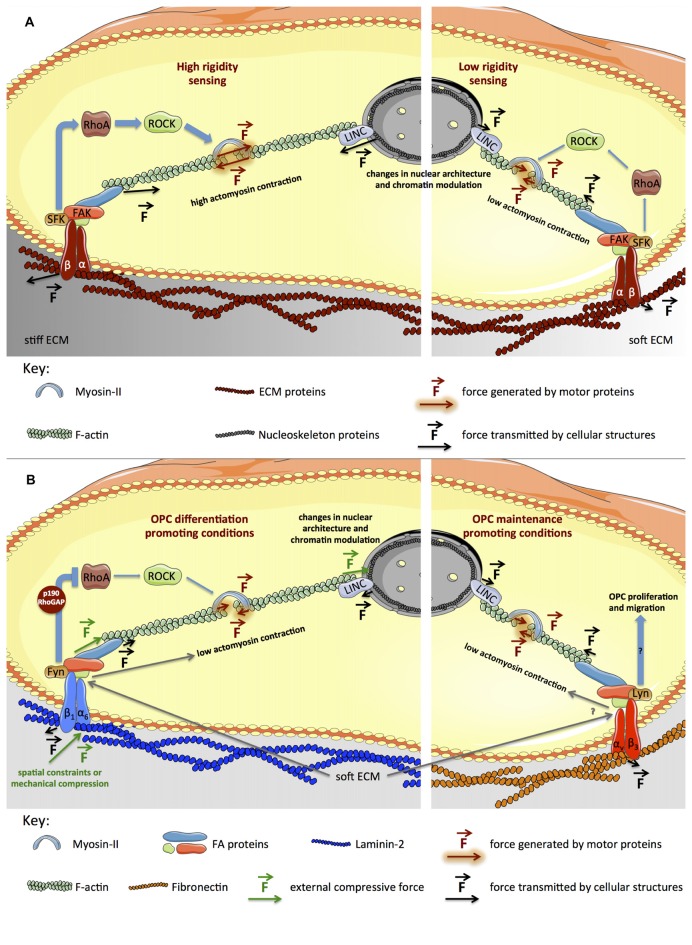
**Signaling pathways generically involved in mechanotransduction and proposed model for the influence of biophysical elements during OL differentiation. (A)** Integrins (heterodimeric transmembrane receptors composed by α and β subunits) engage ECM proteins (Wang et al., 1993) on the extracellular region, in turn recruiting intracellular adaptor proteins that subsequently bind to actin cytoskeleton. Upon integrin activation, several focal adhesion proteins (SFKs, Focal Adhesion Kinase (FAK), Talin) are recruited and activated, promoting cytoskeleton and cellular dynamics (Huveneers and Danen, 2009). On stiffer platforms, focal adhesions (FAs) are reinforced, inducing further activation of RhoA, ROCK and myosin-II, and consequently, cytoskeleton tension increases (left panel). On softer substrates, cytoskeleton tension is lower, due to reduced maturation of FAs and lower activation of RhoA, ROCK and myosin-II (right panel). **(B)** The model presented proposes that inactivation of RhoA caused by activation of Fyn in response to engagement of α6β1 integrin by laminin-2 (Colognato et al., 2004; Bechler et al., 2015) combined with soft substrates contributes to low actomyosin contractility, favoring OL differentiation (left panel). Engagement of αvβ3 integrin by fibronectin leads to activation of Lyn (Colognato et al., 2004), promoting maintenance of OPCs, which also seems to be favored by soft substrates (right panel). Please refer to the main text for further details.

Cells also exert force on the extracellular environment, mostly by action of actomyosin contractility. Tension is transmitted to the extracellular milieu through integrins (Figure 2A), transmembrane heterodimeric receptors linking adherent cells to the extracellular matrix—ECM (Wang et al., 1993). The intensity of cytoskeleton tension produced by adherent cells depends on the cell type, but is also influenced by the physical properties of the ECM of a particular tissue (*in vivo*) or substrate (*in vitro*). It is proportional to the resistance offered by the substrate towards deformation, which in turn is determined by the rigidity (elastic modulus, *E*) of the tissue or material (reviewed in Eyckmans et al., 2011; Sun et al., 2012).

Integrins bind to ECM proteins (e.g., laminin, fibronectin or collagen), providing cellular adhesion (Figure 2A). Although other elements (like the glycocalyx, primary cilia, tight junctions, desmosomes and adherens junctions) also play an important role in mechanotransduction (particularly for fluid shear stress or tissue strain sensing), a central aspect of mechanobiology is the formation of focal adhesions (FAs), which are multi-protein clusters composed by integrins and several intracellular adaptor proteins, that collectively function as a cellular anchor and sensor (Miyamoto et al., 1995; Zimerman et al., 2004). On the intracellular region, adaptor proteins (such as Talin and Vinculin) bind to integrins, in turn recruiting the cytoskeleton (particularly actin filaments). Other proteins associated with FAs, like Focal Adhesion Kinase (FAK) and Src-family kinases (SFKs), regulate Rho-family GTPases, controlling cytoskeleton dynamics, cellular spreading and contractility (reviewed in Huveneers and Danen, 2009). Importantly, FA-associated proteins like p130Cas, Talin or Filamin seem to stretch under tension, in turn exposing further binding sites to FA-adaptor proteins, hence behaving like force sensors. This results in reinforced attachment of actin fibers to FAs and further cellular contractility, with contribution of actin-associated motor proteins like non-muscle myosin-II—NMM-II (Choi et al., 2008; Vicente-Manzanares et al., 2008). This mechanism allows adherent cells to sense forces originating on the extracellular milieu and also to probe the rigidity of the ECM or cell culture substrate (Figure 2A).

The phenotype of mesenchymal stem/stromal cells can be profoundly shaped by biophysical cues, influencing proliferation, differentiation and other aspects of cellular physiology (reviewed in Bellas and Chen, 2014). Typically, conditions that cause low cellular spreading, low actomyosin contractility and low maturation of FAs, such as soft substrates (Engler et al., 2006; Fu et al., 2010), high cell density (Aragona et al., 2013) or spatial constraints (McBeath et al., 2004; Dupont et al., 2011) that prevent cells from fully spreading and exert tension on the ECM/substrate via FAs, result in similar cellular responses. While soft environments and low spreading promote adipogenesis, stiff substrates and cell spreading favor osteogenesis.

Although interactions at the interface between cells or between cells and the extracellular environment (reviewed in Sun et al., 2012; Huveneers and de Rooij, 2013) are at the origin of mechanotransduction signaling, it is known that such stimuli propagate through the cytoskeleton and the linker of nucleoskeleton and cytoskeleton (LINC) complex to the nucleus (Figure 2A), affecting nuclear and chromosomal architecture (Mehta et al., 2010), chromatin modulation (Iyer et al., 2012; Heo et al., 2015; Hernandez et al., 2016) and gene expression (Maier et al., 2008). The discovery of substrate stiffness- and cell shape-responsive transcription factors like YAP/TAZ (Dupont et al., 2011; Low et al., 2014) provided further insights into how gene regulation occurs in response to biophysical cues. Nuclear lamins are also essential for nucleus-mediated mechanotransduction (Swift et al., 2013).

## Mechanobiology of Neural Cells

*In vivo*, cells experience distinct extracellular stiffness and are subjected to different forces, influencing cell fate. When cultured *in vitro*, cells tend to behave more closely to *in vivo* when substrates mimic their native microenvironment (Moore et al., 2010). The elastic modulus of the brain is estimated between 0.1 kPa and 10 kPa, however this broad range of values is still under debate and encompasses results obtained using distinct methods, models and brain regions (extensively reviewed in Chatelin et al., 2010). Moreover, the stiffness of the CNS seems to change with age (Cheng et al., 2008; Clarke et al., 2009; Elkin et al., 2010), although data is somehow contradictory depending on model organism, time points (age) and technique used. Nevertheless, recent studies in living humans, using magnetic resonance elastography, indicate a peak in brain stiffness around adolescence/young adulthood followed by a decline with age (Sack et al., 2011; Arani et al., 2015).

During CNS development, movements and forces are required for the normal formation of brain structures, which also seem to be important for cell-fate specification (Franze, 2013). Neural stem cells (NSCs) differentiate into neurons, astrocytes or OLs *in vitro*, depending not only on the SFs present but also on biophysical elements. Lineage commitment of NSCs is influenced by mechanical cues acting through Rho GTPases, which modulate actomyosin contraction (Figure 2A). High substrate stiffness (1500–75,000 Pa) leads to activation of RhoA and Cdc42 with concomitant intracellular tension and enhanced astrocytic differentiation, whereas inhibition of RhoA and Cdc42 or the presence of a soft culture material (100–700 Pa) favors neurogenesis and oligodendrogenesis (Keung et al., 2011). This strongly suggests prevalence for neuronal and oligodendroglial specification of NSCs induced by soft environments, whereas stiffer ones favor astrocytic commitment.

The neural/neuronal specification of pluripotent stem cells is also influenced by substrate stiffness. The expression of neuroectodermal (Pax6) or neuronal markers (Tuj1) increases when hESCs (or hiPSCs) are cultured on substrates with 100 or 700 Pa, respectively (Keung et al., 2012), indicating that neural progenitors are favored by substrates softer than those promoting neuronal specification.

Lineage specification of NSCs is also affected by tensile strain. Substrate stretching promotes astrocytic and neuronal differentiation while inhibiting oligodendrocytic lineages *in vitro*. Conversely, substrate pre-stretching before cell seeding promoted OL differentiation, with little impact on neuronal or astrocytic commitment. Interestingly, these effects seem to be dependent on specific integrin activation by ECM proteins (Arulmoli et al., 2015). One limitation of this study was that substrate stretching and stiffness could not be uncoupled, i.e., stretched substrates became stiffer than unstretched ones, hence more definitive conclusions as to the real impact of strain during NSC differentiation are difficult to draw.

## Mechanical Modulation of Oligodendrocytes

### Integrin Signaling and OL Differentiation

OLs express a defined integrin repertoire (αvβ1, αvβ3, αvβ5, αvβ8 and α6β1), depending on the cell’s differentiation state and ECM components (Milner and Ffrench-Constant, 1994; O’Meara et al., 2011). Integrin engagement by ECM proteins results in activation of distinct signaling pathways and diverse cellular responses, depending on the ECM protein and integrin repertoires.

Fibronectin activates αvβ3, which recruits the Src-family kinase Lyn, triggering proliferation and survival pathways (Figure 2B), promoting maintenance of the progenitor state of OPCs. Conversely, OL differentiation is promoted by laminin-2/merosin, activating α6β1 and the SFK Fyn (Figure 2B), triggering pathways leading to increased MBP expression and cell maturation (Colognato et al., 2004).

#### Modulation of Rho Family GTPases and the Cytoskeleton

Rho GTPases regulate the polymerization/de-polymerization dynamics of actin, controlling cytoskeleton structures and cellular morphology. Generally, RhoA activation leads to formation of actin stress fibers and actomyosin contractility (Figure 2A), whereas activation of Rac and Cdc42 results in filopodia and lamellipodia formation in several cell types (reviewed in Huveneers and Danen, 2009). In OLs, Fyn activation inhibits RhoA by means of p190RhoGAP (Figure 2B) and promotes Cdc42 and Rac, favoring morphological differentiation (Osterhout et al., 1999; Liang et al., 2004; Laursen et al., 2009; Kramer-Albers and White, 2011). During differentiation, OLs undergo dramatic cytoskeleton rearrangements and consequently morphological alterations, from bipolar to highly branched cells and eventually presenting myelin-rich membranous structures when fully mature—Figure 1 (Bauer et al., 2009; Michalski and Kothary, 2015). OLs comprise two major cytoskeleton components—microtubules and filamentous (F)-actin. F-actin is involved in filopodia and lamellipodia formation in immature OLs, promoting migration. OL maturation occurs with increased morphological complexity, accompanied by enhanced stabilization of microtubules (Bauer et al., 2009; Michalski and Kothary, 2015). Increased morphological complexity is preceded by inactivation of RhoA and consequent decrease of actomyosin contractility, since RhoA activates ROCK (Rho kinase)—the inducer of NMM-II (Figure 2B). Concomitantly, inhibition or abrogation of myosin-II accelerates OL differentiation—leading to increased expression of MBP and cellular branching (Wang et al., 2008, 2012)—and potentiates remyelination after lysolecithin-induced demyelination in adult mice (Rusielewicz et al., 2014).

It was recently observed that F-actin distribution changed dramatically during oligodendroglial differentiation and myelination. During early myelination, F-actin-rich lamellipodia-like protrusions were generated, but subsequently depleted during axonal wrapping and completely disassembled during active myelination (Nawaz et al., 2015; Zuchero et al., 2015). F-actin levels correlate inversely with MBP expression (Zuchero et al., 2015) and membrane tension (Nawaz et al., 2015), hence actin cytoskeleton disruption—caused by a shift of F-actin to G-actin [monomeric/globular (G)-actin]—seems crucial for myelin wrapping.

Arp2/3 (actin nucleation factor) and actin depolymerizing factor ADF/cofilin1 also seem crucial during myelination. Arp2/3 is the major actin nucleation factor, promoting lamellipodia (high F-actin/G-actin ratio), being required during early OL differentiation and initiation of myelination (Zuchero et al., 2015). ADF/cofilin1 is involved in F-actin turnover, contributing to the increased G-actin/F-actin ratio observed in differentiated OLs (Nawaz et al., 2015). The activity of cofilin can be regulated by sequestration to the plasma membrane, namely to phosphatidylinositol 4,5-bisphosphate [PI(4,5)P2]. In mature OLs, MBP competes with cofilin for binding to PI(4,5)P2, releasing cofilin to promote the disassembly of F-actin, hence, contributing to membrane compaction occurring during axonal myelination (Zuchero et al., 2015).

#### Nuclear Modulation and Oligodendrocyte Differentiation

OL differentiation encompasses epigenetic modifications that modulate the genome, silencing genes associated with self-renewal or multi/pluripotency and favoring the expression of others required for terminal differentiation (Hernandez and Casaccia, 2015; Douvaras et al., 2016).

During stem cell differentiation (in general), significant changes occur in nuclear stiffness and architecture in response to mechanical stimuli. This is important for cell fate determination, since the status of sub-nuclear structures, chromatin state and chromosome architecture contribute decisively to the regulation of gene expression (reviewed in Martins et al., 2012). Spatial constraints also affect OL differentiation (Figure 1)—high cell density or the presence of beads with size similar to cells were shown to enhance OPC differentiation. This was attributed to mechanotransduction events encompassing changes in nuclear size and structure, although the mechanistic details were unknown (Rosenberg et al., 2008).

It was recently demonstrated in OLs that compressive forces—either mechanically-driven or due to spatial constraints caused by high cell density or the presence of beads of equivalent size—affect nuclear architecture and chromatin modifications (Figure 2B), causing increased heterochromatic cellular content (Hernandez et al., 2016)—namely increased trimethylated lysine-9 residues of histone-3 (H3K9me), an epigenetic modification associated with OL differentiation and maturation *in vitro* (Douvaras et al., 2016) and *in vivo* (Liu et al., 2012). Concomitantly, increased expression of myelin markers—MBP, CNP and MAG—and axonal myelination were observed. The nuclear changes reported were mediated by the LINC complex (in a *Syne1*-dependent manner) downstream of actin cytoskeleton. In this study, the effect of high cell density and mechanical compression could not be truly uncoupled, since compression also led to indirect increase in cellular density.

## Mechanotransduction and OL Differentiation

During the last decade a growing number of studies revealed the signaling pathways associated with cellular response to forces and other biophysical stimuli and its relevance in cell fate decisions, in particular for OL differentiation and myelination (Figures 1, 2B).

One of the first studies showing the effect of mechanical cues during OPC differentiation used topographical features, mimicking axonal topography and modulating OL alignment and migration (Webb et al., 1995).

More recently, graphene-nanofiber scaffolds were shown to significantly enhance the differentiation of NSCs into OLs (Shah et al., 2014). Nanofibers were used to guide cells, but also to provide spatial cues that mimic axons. Since axonal diameter regulates myelination—thicker axons show increased myelination—nanofibers were created with diameters within the range of axons. Nanofibers with a diameter of 2–4 μm ameliorated the differentiation of OPCs and fiber myelin-ensheathment when comparing with fibers with smaller diameter (<0.5 μm)—Figure 1—recapitulating what is observed *in vivo* (Lee et al., 2012; Bechler et al., 2015). Moreover, the number of myelin sheaths per OL increased in laminin-2-coated fibers through the activation of Fyn pathway (Bechler et al., 2015).

Several studies have focused on the effect of substrate stiffness on OPC fate, beginning to elucidate the molecular pathways involved in such regulatory mechanisms (Kippert et al., 2009; Jagielska et al., 2012; Lourenço et al., 2016). When OPCs were cultured on substrates with a Young’s modulus of ~6 kPa (within the range of the mammalian brain (Chatelin et al., 2010)) the cell surface area of differentiated OLs (a maturation phenotype) was increased in comparison with softer substrates. This effect seemed to be dependent on actomyosin contractility, since its inhibition (using blebbistatin, a NMM-II inhibitor) resulted in a similar effect (Kippert et al., 2009). Later, it was confirmed that OPCs were mechanosensitive and its survival, migration, proliferation and differentiation were influenced by substrate stiffness (Jagielska et al., 2012). Nevertheless, in these studies poly-D-lysine was used to promote cell adhesion (not ECM proteins), hence integrin engagement and the role of ECM proteins were not directly addressed.

Our group showed the importance of combining compliant substrates with ECM proteins for OL differentiation (Lourenço et al., 2016). The maintenance of the progenitor state of rat OPCs was favored by substrates with stiffness similar to rat brain tissues (~6.5 kPa; Juge et al., 2016) functionalized with fibronectin—an ECM protein known to favor the progenitor state of OLs (Colognato et al., 2004). OPC differentiation was improved when cultured on substrates with the same stiffness, but functionalized with laminin-2/merosin (described as promoter of OPC differentiation (Buttery and ffrench-Constant, 1999)), when compared with cells maintained on 6.5 kPa substrates functionalized with poly-D-lysine alone or kept on softer (2.5 kPa) or stiffer (10 kPa or GPa range) substrates. MBP and PLP expression increased and cells displayed a more mature morphology, revealing the importance of combining compliant substrates with ECM proteins for the full maturation of OLs.

In line with our observations, a similar approach was recently published (Urbanski et al., 2016), showing a significant increase in branching complexity of OPCs undergoing differentiation on soft brain-like matrices (1.5 kPa) compared to those kept on rigid substrates (30 kPa; both coated with matrigel), in a NMM-II dependent manner. Differentiation of OPCs on soft conditions led to increased percentage of mature RIP+ and MBP+ OLs and lower nuclear Olig1 content (which translocates to the cytosol during differentiation) when compared to those on stiff substrates. OPCs kept on soft substrates displayed lower nuclear/cytosolic ratio of the mechanosensitive transcriptional regulator YAP when compared to those on stiff substrates (as reported in other cell types (Dupont et al., 2011)), as well as lower nuclear Lamin-A/Lamin-B ratio, previously reported to scale with tissue/substrate stiffness (Swift et al., 2013), hence confirming that OLs are indeed mechanoresponsive cells.

The proposed mechanism for the influence of substrate stiffness during OL differentiation (Figure 2B) is in line with the observation that low RhoA activity and low actomyosin contraction play a positive role in this process (Wang et al., 2008, 2012). Presumably, similar to several cell types (reviewed in Moore et al., 2010; Eyckmans et al., 2011), soft substrates contribute to low actomyosin contraction of OPCs, favoring a low contractile state of the cell, contributing to the differentiation process.

## Conclusion

In this review, the influence of biophysical properties of the ECM and the mechanomodulatory signaling pathways involved in cell fate decisions were discussed, with focus on OL differentiation. OPC fate is affected by proteins of the ECM (or *in vitro* substrates), which engage integrins, activating downstream signaling pathways controlling OL proliferation and differentiation. Recent studies have demonstrated that OPCs are mechanosensitive and its differentiation is influenced by several biophysical cues. Nevertheless, the pathways involved in the conversion of mechanical forces into biochemical signals during OL differentiation remain partially elusive, requiring further mechanistic studies that will eventually contribute to a better understanding of myelination/remyelination processes.

## Author Contributions

TL and MG wrote and revised the manuscript and created the figures. Both authors approved the final version of the manuscript for publication.

## Funding

Authors acknowledge funding by the ERDF through Programa Operacional Factores de Competitividade—COMPETE and by national funds by FCT—Fundação para a Ciência e a Tecnologia (Portuguese Foundation for Science and Technology) through grants FCOMP-01-0124-FEDER-021150-PTDC/SAU-ENB/119292/2010 attributed to MG, which included a research fellowship awarded to TL, and COMPETE funding (Project “Stem cell based platforms for Regenerative and Therapeutic Medicine”, Centro-07-ST24-FEDER-002008).

## Conflict of Interest Statement

The authors declare that the research was conducted in the absence of any commercial or financial relationships that could be construed as a potential conflict of interest.
